# Philadelphia County Medical Society

**Published:** 1888-08

**Authors:** 


### Philadelphia County Medical Society.

*Stated Meeting, April,* 25, 1888.

THE PRESIDENT J. SOLIS-COHEN, M.D., IN THE CHAIR.

[Dr. George McClellan reported a case of amputation of the left half of the Tongue for Epithelioma.]

Michael G., an Irishman, aged 40, was admitted the first of this month to my ward at the Philadelphia Hospital. He stated that he had never had syphilis, and that his general health had always been good—which was borne out by his appearance. He had always been an intemperate smoker of a short-stemmed pipe. Six months ago he first experienced sharp and shooting pain extending from the root of the tongue on the left side down the neck and over the face. A small sore was noticed on the side of the tongue about its middle, which rapidly increased until, at the time of admission to the hospital, it was the size of a half dollar. Articulation and deglutition were interfered with, and there was a constant flow of saliva. There was also, apparently, an enlarged gland in the submaxillary triangle. I diagnosed the disease to be epithelioma, and undertook its removal by amputation on April 11, just two weeks ago. An incision was made from the symphysis of the chin, a finger's breadth below the jaw as far as the external jugular vein; the deep cervical fascia was torn through with the fingers and knife handle, and the swelling, which was under the sternomastoid muscle, proved to be a degenerated gland, or rather cyst, which was filled with thick cheesy matter. The cyst was evacuated of its contents, the cyst wall torn out as much as possible, and the lingual artery, which was exposed in its relation to the hyoid bone, the hypo-glossal nerve, and digastric muscle, was secured by a ligature. The anterior belly of the digastric was severed, and the mylohyoid muscle with the oral mucous membrane punched through with the finger, and the tongue having been freed from its frænum and pierced at its apex with a strong needle and string, was pulled down into the wound in the neck, as I hoped by so doing to be able to take away the diseased portion by the first incision. This proved unadvisable, owing to the short, thick neck of the patient, and would have endangered the carotid and deep vein, and I at once cut through the commissure of the mouth, and after tying the coronary arteries, pulled the tongue forward, passed a trocar and canula through the middle of its base close to the hyoid bone, the trocar was withdrawn, and the chain of an ecraseur was passed through the canula, and, after withdrawing the latter, I found the ecraseur worked admirably. I then cut off the affected half of the tongue close to the raphe, and was able to show my assistants that the only bleeding vessels from the tongue itself were at its apex. A ligature secured these, and the stump was lightly touched with the Paquelin cautery. Both wounds were then united by interrupted silk sutures and dressed antiseptically. There was a rise of temperature to 103° the evening following, which was reduced by quinine suppositories and sponging. Since then it has been about normal. There was a great deal of venous bleeding during the operation, but very little arterial, owing to the early and prompt securing of the lingual and branches of the facial. Cracked ice and iced milk only were allowed the patient for the first twenty-four hours. The wounds were found healed at the first dressing on the third day, and this morning, (April 25), I saw the patient sitting up in a chair, dressed and wishing to go out. He has had no pain whatever, and talks perfectly, and takes nourishment better than for a long time before the operation.

*Dr. G. G. Davis:* I would like to ask Dr. McClellan where he tied the lingual artery. It is usually taught that the proper place to expose this vessel is in the digastric triangle. My own experience, in teaching the operation upon the cadaver, is that it may often be more readily found and ligated behind the digastric, thus obviating the necessity of cutting through the mylohyoid muscle. In the hip-joint excision it would be interesting to know how far down the shaft of the bone was removed. If a preliminary resection of the joint and shaft low down is practiced, and afterward an amputation through the thigh performed, I

believe that the operation will be of much less gravity than if the whole operative procedure is undertaken at one time. Of course this refers only to cases in which the condition of the patient is such as to make a primary hip-joint amputation too dangerous.

*Dr. McClellan:* The lingual artery is not difficult to secure. The land-mark is the great cornu of the hyoid bone, and by making a curved incision parallel to the angle of the lower jaw, over it, we can readily expose the looped tendon of the digastric muscle and the stylo-hyoideus. Here the hypo-glossal nerve is found, and the lingual artery passes immediately behind it.

It will be remembered that there was a great mass in the neck, probably due to secondary involvement from the growth on the tongue. The first incision was made with the view of removing the gland and bringing out the tongue laterally by the same means of access. The gland was found beneath the sterno-mastoid muscle, and a dissection to expose it would have endangered not alone the carotid, but the deep jugular, which I am loath either to wound or to tie. I therefore tore out the cyst after discharging its contents, and then brought into view the parts over the lingual.

In looking over the history of excision of the tongue, I have been struck with the fact that the different methods adopted, all have points worthy of consideration in different cases. The operation of cutting through the symphysis is sometimes practiced, also the horseshoe sub-mental incision. The lateral operation is applicable only in a long, thin neck. It could not be performed in a short thick neck. The other operation of simply cutting through the oral commissure is very easy and simple, and exposes the parts sufficiently in all cases where the disease is situated behind the middle of the organ. If we simply remember that the deep cervical fascia which loops down the two bellies of the digastric muscle also covers the artery and nerve, we need have no difficulty in finding the lingual, and it is readily distinguished from the nerve which lies in front of it, both upon the cadaver and living patient. The difficulties sometimes experienced in operating on the former are due to the relation of the parts not being properly remembered.

